# Carbanion as a Superbase for Catalyzing Thiol–Epoxy Photopolymerization

**DOI:** 10.3390/polym9090400

**Published:** 2017-08-29

**Authors:** Xiaoqing Dong, Peng Hu, Weizhen Shen, Zhiquan Li, Ren Liu, Xiaoya Liu

**Affiliations:** 1 Key Laboratory of Synthetic and Biological Colloids, Ministry of Education, School of Chemical and Material Engineering, Jiangnan University, Wuxi 214122, Jiangsu, China; dongxiaoqing@aqnu.edu.cn (X.D.); 6160608012@jiangnan.edu.cn (P.H.); 6140610043@jiangnan.edu.cn (W.S.); liuren@jiangnan.edu.cn (R.L.); lxy@jiangnan.edu.cn (X.L.); 2 Collaborative Innovation Centre for Petrochemical New Materials, AnHui Province Key Laboratory of Optoelectronic and Magnetism Functional Materials, School of Chemistry and Chemical Engineering, Anqing Normal University, Anqing 246013, Anhui, China

**Keywords:** photopolymerization, thiol–epoxy, carbanion, superbase, decarboxylation, thioxanthone

## Abstract

Photobase generator (PBG)-mediated thiol–epoxy photopolymerization has received widedspread attention due to its versatility in various applications. Currently, nearly all reported PBGs release amines as active species. The formed amines induce odor, yellowing, and potential toxicity. In this study, a series of novel thioxanthone-based PBGs, which were able to generate carbanion via decarboxylation under LED light irradiation, were designed and straightforwardly prepared. The formed carbanion can be used as a superbase to catalyze thiol–epoxy polymerization efficiently. Investigation on ^1^H NMR and FT-IR confirmed the generation of carbanion intermediates. The counteranion significantly affected the photodecarboxylation efficiency. The study of photopolymerization tests, based on real-time FT-IR and dielectric analysis measurements, indicated that the generated carbanion exhibited faster polymerization rate and higher epoxy conversion than traditional superbase 1,8-diazabicyclo[5.4.0]undec-7-ene (DBU). In differential scanning calorimeter, thermogravimetric, and nanoindentation tests, comparable thermal and mechanical properties of the photocured films catalyzed by novel PBGs were obtained. The high initiation ability combined with straightforward synthesis makes these PBGs promising candidates for commercialization.

## 1. Introduction

The “click” reaction has attracted widespread public attention in materials science. Such versatile chemistry features benign reaction conditions, a high reaction rate, high yields, and outstanding orthogonal reactivity [1,2]. Among the family of click reactions, the base–catalyzed thiol–epoxy reaction, which provides materials with reduced shrinkage and excellent mechanical properties, has been widely employed in various applications, including photoresists, imaging, adhesives, and high-performance coatings [3,4,5]. Photoinduced thiol–epoxy polymerization possesses additional advantages of spatial and temporal control of the process [6]. A highly-active photobase generator (PBG) is essential to obtaining an efficient photoinduced thiol–epoxy photopolymerization [7,8,9]. However, it remains challenging to develop ideal PBGs that generate active species with high quantum yields under irradiation and contain a suitable chromophore for matching the irridiation source.

A variety of PBGs, such as acyloxyimines, oxime–urethanes, and α-keto carbamates, have been reported [10,11,12]. However, long exposure time and high post-baking temperatures are needed due to the weak alkalinity of the formed active species after irradiation. Recently developed PBGs containing superbase, like DBU (1,5-diazabicyclo[5.4.0]undec-5-ene) and TBD (1,5,7-triazabicyclo[4.4.0]dec-5-ene), can provide efficient polymerization without post-baking [8,13,14,15]. In order to extent the absorption wavelength from UV-C and UV-B range (<320 nm) to UV-A and visible region, a hybrid photoinitiation system based on a physical mixture of a photolatent PBG and isopropylthiolxanthone (ITX) as a visible light photosensitizer has been proposed [16]. Excitation under visible light has some significant advantages in irradiation safety, energy utilization efficiency, and deeper curing depth [17]. The limitation of such a photoinitiating system derives from the intrinsic drawback of bimolecular systems, where the back electron transfer occurs between the excited photosensitizer and the PBG. Such a process would reduce the quantum yield of the active species [18]. Recently, Bowman et al. reported several unimolecular coumarin-based PBGs [19]. These PBGs exhibited extraordinary catalytic activity toward initiation of the thiol–Michael reaction upon visible-light irradiation. Our group also developed a series of unimolecular thioxanthone–based PBGs [20], which can release superbase via photoinduced decarboxylation to catalyze thiol–epoxy photopolymerization.

Currently nearly all reported PBGs release amines as active species under irradiation. However, the efficient PBGs containing super bases such as TBD and DBU usually exhibit limited solubility in thiol–epoxy resin due to the rigid ring of the amine [8,13]. Moreover, after photopolymerization, the residue amine would induce some unwanted reactions to affect the property of the cured materials, such as oxidation, to yield yellowing. Therefore, to explore alternative active species is of particular interest. Recently, Wan et al. demonstrated that carbanion can play a role of base to induce anionic polymerization of acrylates [21]. The carbanion derived from ketoprofen (KP) derivatives after photodecarboxylation. Their work inspired us to explore whether the active carbanion can be used as a promising alternative to efficiently induce thiol–epoxy polymerization without the abovementioned disadvantages caused by using amine-based PBGs.

**Figure 1:**
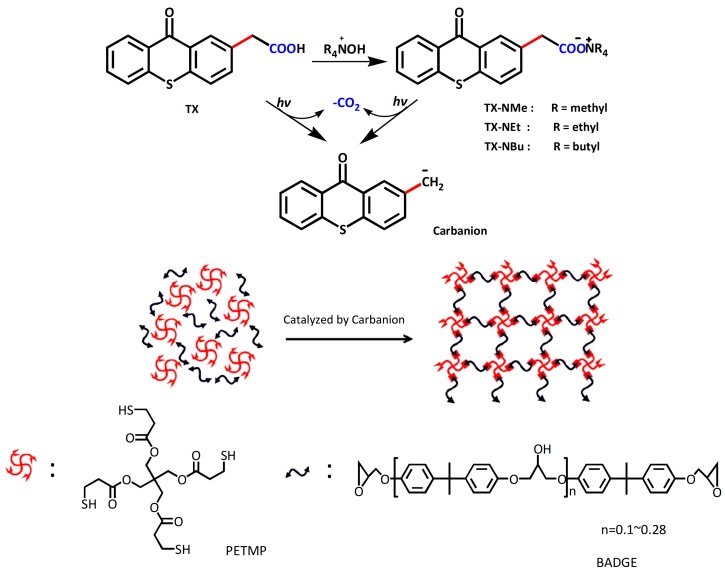
Structures of the PBGs designed to generate carbanion and their application in thiol–epoxy polymerization.

In this study, we report a series of thioxanthone-based PBGs which are able to generate carbanion after photoinduced decarboxylation (Figure 1). The photochemical behaviors of the PBGs were investigated and the released active basic species were confirmed. The ^1^H NMR measurements were performed to evidence the carbanion intermediates in the decarboxylation reaction, and the ability of the carbanion in ring-opening polymerization was also confirmed by FT-IR and ^1^H NMR measurements. Moreover, photopolymerization kinetics were studied using real-time FT-IR and photodielectric analysis to verify the activity of the PBGs in initiating thiol–epoxy polymerization. Finally, differential scanning calorimeter, thermogravimetric, and nanoindentation analysis were used to study the thermal and mechanical properties of the photocured films. For a comparison, a previously reported PBG containing DBU [20] was also tested.

## 2. Materials and Methods

### 2.1. Materials

Thiosalicylic acid and tetraethyl ammonium hydroxideand were purchased from Aladdin Industrial Corporation (Shanghai, China). Pentaerythritol tetra(3-mercaptopropionate) were supplied by TCI Chemicals Pvt. Ltd. (Shanghai, China). Triphenylphosphine, sulfuric acid, tetramethylammonium hydroxide, tetrabutylammonium hydroxide, and the other solvents were bought from Sinopharm Chemical Reagent Co., Ltd. (Shanghai, China). The epoxy monomer (BADGE, a diglycidyl ether) were purchased from Jiangsu Sanmu Group (Yixing, China). All the chemicals were used as received without further purification.

### 2.2. Characterization

^1^H NMR and ^13^C spectrum were measured using a Bruker instrument (AVANCE III HD 400 MHz, Billerica, MA, USA). UV–Vis spectra were measured with a Beijing Purkinje TU-1901 UV–Vis spectrophotometer (Beijing, China). The incident light intensity was monitored by power meter (UV Power Puck II from EIT Sterling, VA, USA). The UV Pro YW-51220 LED lamp (Shanghai UV Pro Co., Ltd., Shanghai, China) and IWATA UV-100 LED (Shanghai, China) irradiate 365 nm light. The polymerization experiments were carried out using real-time FT-IR with a Nicolet 6700 FT-IR spectrometer (Waltham, MA, USA) worked with an OmniCure Series 1000 UV spot curing system (a high-pressure mercury lamp, spectral emission: 320–500 nm, maximum 365 nm, Waltham, MA, USA), which adjusted the incident light intensity according to the monitor of the EIT power meter. High-resolution mass spectrometer measurements were performed using Waters MALDI SYNAPT Q-TOF MS (Milford, MA, USA).

### 2.3. Photolysis

The changes of the UV–Vis spectra during photolysis were recorded in acetonitrile with different light doses. The light source was a UV Pro YW-51220 LED lamp of 365 nm light, and the light intensity was measured by to the EIT Power Puck II. A very small stirrer was kept stirring in the cuvette. A drop of saturated phenol red acetonitrile solution was added in the cuvette to detect the released basic species.

### 2.4. Real-Time FT-IR

Real-time FT-IR spectroscopy was used to monitor the epoxy band absorption as a function of exposure of irradiation in the resins. An Hg lamp adapted to the FT-IR spectrometer by means of a light guide was used to adjust the light intensity to 18.4 mW/cm^2^ and to irradiate the resins. PBG (2 mol %) was added to the corresponding monomers and well-mixed by ultrasonic vibration for 10 min (Thioxanthone (TX) was dissolved in 0.3 mL acetone firstly). Before the photopolymerization, the mixture was dried under vacuum for 30 min at room temperature in order to remove the solvent. The thickness of the mixture film was adjusted by a 120 μm film applicator (BYK, Wesel, Germany). Polymerization profiles were recorded during 1000 s irradiation at room temperature. For each sample, the measurements were tested more than three times. The polymerization kinetics were measured by monitoring the disappearance of thiol and epoxy band, and the conversion was calculated using the formula:

(1) $$Conversion\;(\%)=[1-\frac{A_{t}}{A_{0}}]\times 100\%$$

where *A_t_* is the area of the epoxy and thiol characteristic absorbance peak at 915 and 2568 cm^−1^ at time *t*, and *A*_0_ stands for the initial area of this peak.

### 2.5. Dielectric Analysis

The dielectric analysis (DEA)-measurements (NETZSCH, DEA288, Freistaat Bayern, Germany ) were performed with a dielectric analyzer using Mini-IDEX sensors and an OmniCure UV spot curing unit. Highly-viscous resin was placed on the sensor and data collection from the DEA analyzer was started to determine the ion viscosity of the resin. Typically after 60 s the light was switched on starting the curing process until a saturation level was reached. For each sample, the measurements were tested three times. A frequency of 1 Hz was chosen in the experiments at a temperature of 25 °C due to the slow curing process of these resins cause by the low light intensity (1.95 mW/cm^2^) to avoid the thermal effect derived from the irradiation source.

### 2.6. Thermal Properties Measurements

The resin formulations applied in real-time FT-IR measurements were irradiated under a 365 nm LED lamp with an output power of 1.1 W/cm^2^ for 8–10 min. The thermal properties of cured films were studied by differential scanning calorimeter (DSC) and thermogravimetric analysis (TGA). Cured films were analyzed by a PE DSC-8000 (Shanghai, China) under a constant N_2_ atmosphere with a gas flow of 100 mL/min, which calibrated using an indium standard (heat flow calibration). Sample weights were 10.0 ± 0.5 mg. The glass transition temperature (*T*_g_) of the cured films were determined in a dynamic scan at 30 °C /min in the temperatures range of 30–300 °C.

TGA were carried out with a Mettler TGA/DSC/1100SF (Columbus, OH, USA). Pieces of the cured films with a mass of 10.0 ± 0.5 mg were degraded between 25 and 650 °C at a heating rate of 20 K/min, under an inert atmosphere (N_2_ at 50 mL/min).

### 2.7. Nanoindentation Tests

The mechanical properties of cured materials were conducted with a nanoindentation system (Nanoindenter G200, Agilent Technologies Inc., Santa Clara, CA, USA). A Berkovich indenter tip with a radius of curvature of 50 nm was employed for indentation experiments. The indentation was measured with a constant force of 500 μN to test the hardness and reduced modulus. The loading time, dead loading time, and discharge time were 5 s; the distance between every point was 50 nm. For each sample, the measurements were tested five times. The thickness of the photocurable films was adjusted by a 120 μm film applicator (BYK) and irradiated under a LED lamp with an output power of 1.1 W/cm^2^ for 8–10 min.

### 2.8. Synthetic Procedures

Considering that the thioxanthone derivatives are photosensitive to visible light, the following preparation were performed in a yellow light room and the glassware were wrapped with tinfoil.

#### 2.8.1. Synthesis of Thioxanthone Acetic Acid (TX)

Thiosalicylic acid (1.54 g, 10 mmol) was slowly added to 20 mL of concentrated sulfuric acid and the mixture was stirred for 30 min to ensure through mixing. Phenylacetic acid (4.05 g, 30 mmol) was slowly added to this stirred mixture. After the addition, this mixture was fiercely stirred for 2 h at room temperature and then for 2 h at 75 °C. Then the mixture was cooled to room temperature and stirred overnight. Afterward, this mixture was slowly poured into water (200 mL) with stirring, and precipitation was collected and washed with water to afford a crude blue solid, which was recrystallized three times from dioxane/water mixture to give the product as a green solid (0.52 g, yield of theory: 18%). Although there was residual 3-thioxanthone acetic acid, the product was directly used in the next step without further purification. ^1^H NMR (400 MHz, CD_3_CN) *δ*, *ppm* 9.25 (s, 1H), 8.58–8.34 (m, 2H), 7.74–7.54 (m, 5H), 3.82 (s, 2H).

#### 2.8.2. Synthesis of Compound (9-Oxo-9H-Thioxanthen-2-yl)-Acetatetetraethyl-Ammonium (TX–NEt)

The solution of tetraethylammonium hydroxide in H_2_O (0.57 mL, 1 mmol, 25 wt %) was added to TX (0.295 g, 1.1 mmol). After the powder of TX was quickly dissolved, the solution was filtrated to remove the overdose of TX and evaporated under vacuum at room temperature to yield the dark red solid (Yield of theory: 95%). ^1^H NMR (400 MHz, CD_3_CN) *δ*, *ppm* 8.61–7.55 (m, 7H), 3.51 (s, 2H), 3.24 (q, *J* = 7.2 Hz, 8H), 1.26 (t, 12H). ^13^C NMR (101 MHz, DMSO-*d*_6_) *δ*, *ppm* 180.03, 179.41, 140.62, 137.20, 135.38, 134.68, 133.22, 133.12, 129.54, 129.37, 129.25, 128.93, 128.23, 127.16, 126.97, 126.92, 126.27, 125.86, 51.90, 51.87, 51.84, 47.16, 7.54. Q-Tof-MS (*m*/*z*): calcd for C_8_H_20_N^+^, 130.1596. Found: 130.1602 [M]^+^.

#### 2.8.3. Synthesis of Compound (9-Oxo-9H-Thioxanthen-2-yl)-Acetatetetramethyl-Ammonium (TX–NMe)

Using a similar preparation manner to that of TX–NEt, the solution of tetramethylammonium hydroxide in H_2_O (0.36 mL, 1 mmol, 25 wt %) was added to TX (0.295 g, 1.1 mmol). After the powder of TX was quickly dissolved, the solution was filtrated to remove the overdose of TX and evaporated under vacuum at room temperature to yield the viscous oil (Yield of theory: 94%). ^1^H NMR (400 MHz, DMSO-*d*_6_) *δ*, *ppm* 8.58–7.44 (m, 7H), 3.30 (s, 2H), 3.10 (s, 12H). ^13^C NMR (101 MHz, DMSO-*d*_6_) *δ*, *ppm* 179.42, 137.19, 135.30, 134.72, 133.13, 129.51, 129.41, 129.37, 129.25, 128.90, 128.26, 127.14, 126.92, 126.04, 125.95, 54.82, 54.78, 54.74, 46.88. Q-Tof-MS (*m*/*z*): calcd for C_4_H_12_N^+^, 74.0970. Found: 74.0982 [M]^+^.

#### 2.8.4. Synthesis of Compound (9-Oxo-9H-Thioxanthen-2-yl)-Acetatetetrabutyl-Ammonium (TX–NBu)

Using a similar preparation manner to that of TX–NEt, the solution of tetrabutylammonium hydroxide in H_2_O (2.6 mL, 1 mmol, 10 wt %) was added to TX (0.295 g, 1.1 mmol). After the powder of TX was quickly dissolved, the solution was filtrated to remove the overdose of TX and evaporated under vacuum at room temperature to yield the viscous oil (Yield of theory: 92%). ^1^H NMR (400 MHz, DMSO-*d*_6_) *δ*, *ppm* 8.86–8.13 (m, 2H), 7.82–7.34 (m, 5H), 3.30 (s, 2H), 3.17–3.09 (m, 8H), 1.57–1.48 (m, 8H), 1.37–1.27 (m, 8H), 0.91 (t, *J* = 7.3 Hz, 12H). ^13^C NMR (101 MHz, DMSO-*d*_6_) *δ*, *ppm* 175.36, 170.75, 134.17, 132.82, 132.06, 130.17, 129.60, 129.12, 127.25, 124.98, 124.86, 124.64, 124.47, 124.32, 123.88, 123.79, 122.27, 121.61, 121.57, 121.29, 121.18, 120.76, 72.64, 72.52, 72.32, 72.00, 53.80, 41.04, 19.08, 14.85, 8.83. Q-Tof-MS (*m*/*z*): calcd for C_16_H_36_N^+^, 242.2848. Found: 242.2840 [M]^+^.

## 3. Results and Discussion

### 3.1. Synthesis of Photobase Generators

Several studies have proven that both the deprotonated KP and the acidic form of KP can undergo photodecarboxylation to generate carbanion [22,23,24], which can play the role of the base to induce anionic polymerization of acrylates [25,26]. Since the investigated PBG (TX) has a similar structure to KP, similar photodecarboxylation mechanism and active intermediate carbanions can be expected. Substation of benzophenone by thioxanthone can red-shift the absorption band to the visible light region. The synthesis route of TX has been well developed [27]. Some studies have proven that dissociated KP underwent a substantially faster decarboxylation reaction than did undissociated KP [28,29]. To study the effect of counterions on the photodecarboxylation rate, quaternary ammonium salts of TX were straightforwardly prepared via neutralization between TX and corresponding ammonium hydroxides with high yields (Figure 1). The prepared quaternary ammonium salts exhibit improved solubility in thiol–epoxy formulations compared to the previously-reported PBG TX–DBU containing the rigid ring of the amine. For TX–DBU, the maximum concentration of the initiator is 2 mol %, while for the novel PBGs containing different quaternary ammonium salts, the corresponding concentration can reach more than 10 mol %.

### 3.2. Photolysis Study

**Figure 2:**
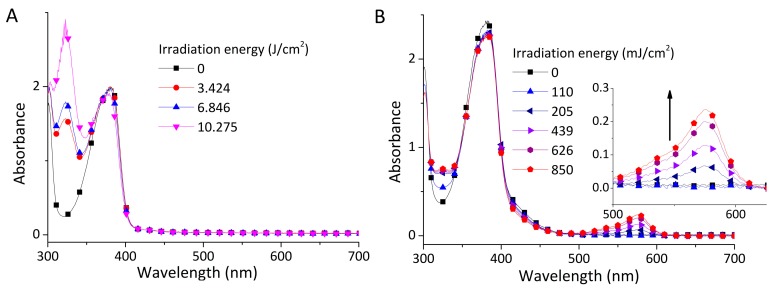
UV–Vis spectra of the TX solution (10^−4^ M) without (**A**) and with (**B**) the addition of phenol red under irradiation at different light doses.

The UV–Vis absorption spectra of the PBGs were recorded in acetonitrile (Figure S1). The results of photolysis conducted upon irradiation by an LED lamp emitting 365 nm light are shown in Figure 2. The shape and the wavelength of the absorption peaks were quite consistent because of thioxanthone derivatives possessing the same chromophore [20]. The characteristic absorption peaks at 385 nm attributed to the *n*-π* transition did not obviously change. A new peak emerging at 323 nm under irradiation was assigned to the absorption of ketyl radical (Figure 2A) [30]. Furthermore, as displayed in Figure 2B, basic species were detected under irradiation when one drop of phenol red was added as a general pH indicator. Increasing the dose of light increased the characteristic absorption peak at 575 nm, which was assigned to the deprotonated phenol red after reaction with the basic–species (Figure 2B). A similar colorimetric method was applied to evidence the formation of free bases by Arimistu [8] and Allonas [13]. TX–NEt behaved in a similar manner except for no new peak emerged in the range of 323 nm (Figure S2).

To evidence the photoinduced decarboxylation reaction of the PBGs, a common procedure described elsewhere was performed to detect the generated CO_2_ [13]. *N*,*N*-dimethylformamide solutions of TX–NEt (Figure S3) and TX were separately placed in sealed bottles which were connected to another bottle containing an aqueous solution of Na_2_CO_3_ with one drop of phenolphthalein. The decarboxylation occurred under the irradiation of LED lamps, which induced the released CO_2_ to diffuse through the connecting tube and neutralize the Na_2_CO_3_ in the other bottle (Figure S3). Bubbles were observed in the bottle containing the aqueous solution and the pink color disappeared after 30 min of irradiation. The results confirmed the release of CO_2_ though a photoinduced decarboxylation reaction.

**Figure 3:**
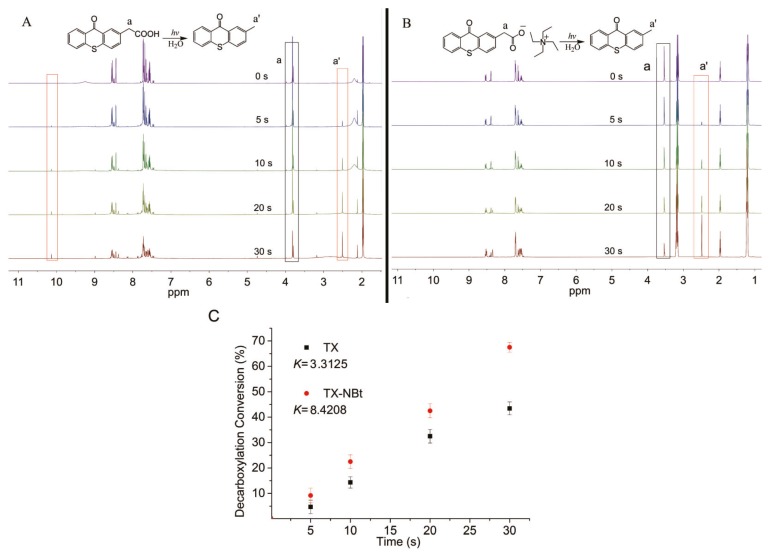
Photodecarboxylation ^1^H NMR spectra (CD_3_CN) of TX (**A**) and TX–NEt (**B**) under an LED lamp with an output power of 1.1 W/cm^2^; decarboxylation conversion (DC) of TX and TX–NEt under irradiation (**C**). K stands for NMR-observed rate constants [34]. DC was calculated by the integration area of methylene at 3.6 ppm.

Since the preceding CO_2_ detection experiment provided only qualitative information, ^1^H NMR data were collected to determine instantaneous concentrations of detectable photolysis species after photodecarboxylation (Figure 3). The conversion degree of decarboxylation was calculated by comparing the integration changes of methylene peaks [31,32]. A substantial change in the ^1^H NMR spectra were observed under irradiation. The characteristic peak of methylene decreased concomitantly with a new peak emerging at 2.5 ppm, which was assigned to the methyl group of methylthioxanthone [33]. The photodecarboxylation of TX–NEt was considerably faster than that of TX (Figure 3C). Moreover, irradiation of TX led to a new peak emerging at 10.15 ppm, suggesting that ketyl radicals were produced under irradiation through a hydrogen transfer process (Figure 3A). By contrast, no new peaks appeared in this range for TX–NEt after irradiation (Figure 3B).

Additional NMR analysis was executed to evidence the carbanion intermediates as described by Scaiano et al. [22]. One drop of D_2_O was added to the CDCl_3_ solution of TX–NEt, which was irradiated and analyzed by ^1^H NMR. Irradiation of the solution provided the corresponding α-deuteromethylthioxanthone with an amount of methylthioxanthone owing to the presence of residual H_2_O. Based on the characteristic triplet of the –CH_2_D signal, which was slightly upfield from the –CH_3_ signal, the α-deuteromethylthioxanthone was easily determined and integrated for two hydrogens relative to the aromatic signals of deuteromethylthioxanthone (Figure S4). Since D_2_O is not an effective deuterium atom donor but an excellent D^+^ source, the results demonstrated that the PBGs efficiently generated carbanion intermediates through photodecarboxylation reactions [22].

### 3.3. Carbanion-Catalyzed Ring-Opening Polymerization

**Figure 4:**
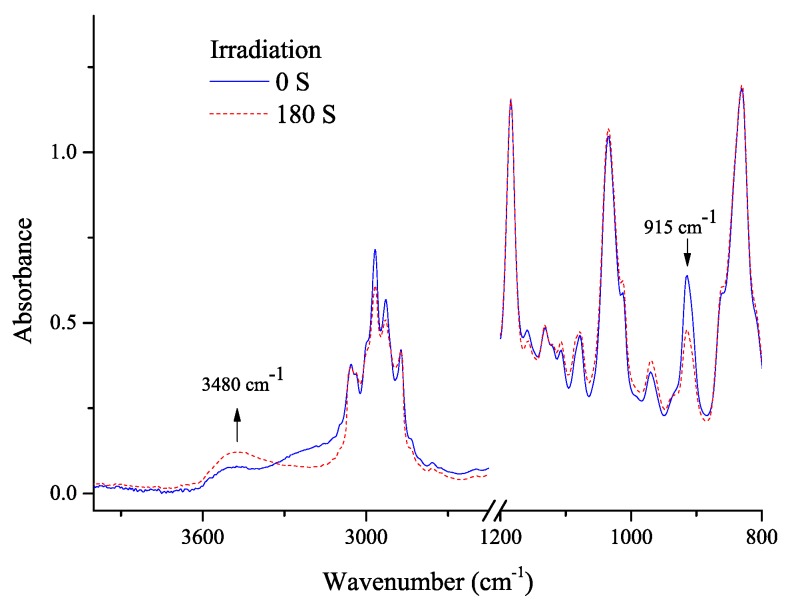
Transmission FT-IR spectra of BAGDE (in the presence of TX) under UV irradiation. Sample preparation conditions: sandwiching the mixture between two KBr salt plates and UV irradiation (1.0 W cm^−2^).

Carbanion had been proved to initiate anionic polymerization [35]. Therefore, it is of interest to explore to explore whether the carbanion can directly induce ring-opening of epoxy in the absence of thiol monomers. A mixture, containing of TX (10 mol %, dissolved in 0.5 mL of DMF) and BADGE (90 mol %), was irradiated and analyzed using FT-IR. As displayed in Figure 4, the decreasing singlet at 915 cm^−1^ assigned to the C–O asymmetrical stretching of epoxy indicated the consumption of the epoxy groups. FT-IR spectra before and after the irradiation showed a marked modification of both the ring-opened and non-opened epoxy groups. Accordingly, the broad singlet at 3480 cm^−1^ assigned to the O–H stretching of hydroxyl increased [36].

Additional ^1^H NMR tests were employed to prove the ring-opening polymerization of the epoxy resin initiated by the carbanion. 10 mol % TX and 90 mol % of BAGDE were dissolved in 0.5 mL of DMSO-*d*_6_). After irradiation, the methine peak of the epoxy ring at 3.30 ppm decreased, while the peak of 4.01 ppm assigned go the methine after thering-opening reaction [37] increased (Figure S5A). Ring-opening conversion of BADGE, calculated by integrating for the methine of the epoxy ring, was shown as a quantitative description of ring-opening polymerization (Figure S5B). The combined results from FT-IR and NMR showed that the carbanion could initiate the ring-opening polymerization of epoxy resin.

### 3.4. Carbanion-Catalysed Thiol–Epoxy Photopolymerization

After confirming the ring-opening polymerization of epoxy resin initiated by the carbanion, we studied the ability of the carbanion to initiate thiol–epoxy polymerization. Real-time FT-IR measurements were performed to study the photopolymerization kinetics. A photosensitive formulation, containing a mixture of PBGs (2 mol %), pentaerythritol tetra (3-mercaptopropionate) (PETMP, 49 mol %), and epoxy (BAGDE, 49 mol %), was irradiated under a UV-A LED lamp (365 nm). For a comparison, a previously-reported highly-efficient PBG (TX–DBU) was also tested.

**Figure 5:**
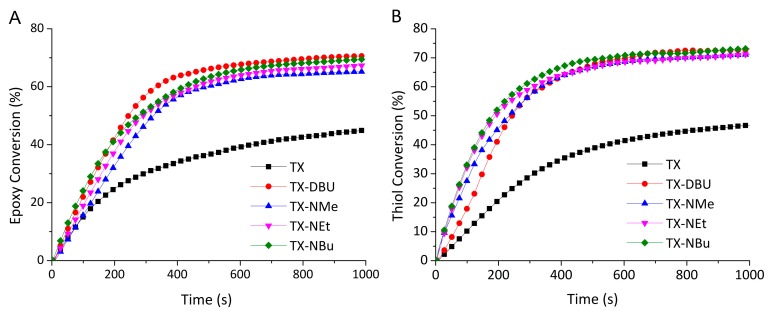
Conversion curves for photopolymerization initiated by the PBGs.

All investigated PBGs can induce thiol–epoxy polymerization under irradiation without post-baking. TX exhibited the slowest polymerization speed and lowest final epoxy conversion among the investigated PBGs (Figure 5). The PBGs containing quaternary ammonium cations exhibited a faster polymerization rate, as well as higher final epoxy conversion, similar to those of the superbase-released TX–DBU. As shown in the photolysis tests, TX–NEt exhibited a higher efficiency in the decarboxylation reaction than TX did. Fast photodecarboxylation can provide rapid generation of the active carbanion for initiating polymerization. Other quaternary ammonium salts behaved in a similar manner. The results revealed that using the quaternary ammonium salts greatly enhanced the polymerization rate, as well as final epoxy conversion, owing to the efficient generation of the carbanion.

TX–NBu exhibited the best performance in both the polymerization rate and epoxy conversion. This behavior may be explained by the relatively higher electronic effect and steric hindrance of the butyl groups. Tetraalkylammonium cations are a particularly useful bridge for facilitating the electron transfer process in photoinduced polymerization reactions [38]. This process also influences the triplet state lifetime of PBGs.

Although the decarboxylation mechanism is very complex and several issues are not yet clear, it is believed that decarboxylation is followed by deprotonation. Dissociated KP undergoes a substantially faster decarboxylation reaction than does undissociated KP [29], suggesting that the undissociated TX undergoes an extra–deprotonation process. The current study on laser flash photolysis measurements confirmed that transient absorption differed between dissociated and undissociated TX, for which the transient spectroscopy of the triplet state showed two peaks at 630 nm unlike the single peak of TX–NEt (Figure S6). Furthermore, the ketyl radical and decarboxylation formation of TX were observed through ^1^H NMR (Figure 3), and were confirmed according to the UV–Vis absorption spectrum (Figure 2A). Due to the slow decarboxylation, the performance of TX was inferior to that of the other PBGs that contained quaternary ammonium cations [29].

**Figure 6:**
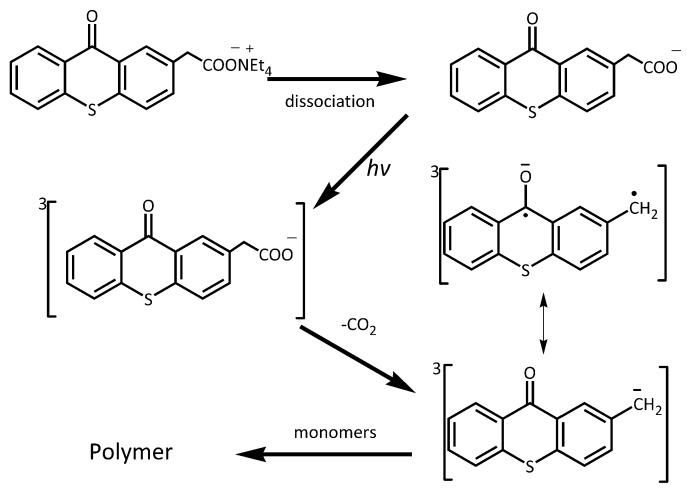
The proposed photoinitiated polymerization mechanism of the thioxanthone derivatives.

TX has been demonstrated as a free radical photoinitiator by Yagci [39] and Arsu [27]. The electron spin resonance (ESR) measurement of TX–NEt and TX were provided in Supporting Information (Figure S8). The ESR signal demonstrated that both of the PBGs generated the same free radicals, owing to the similar decarboxylation reactions. In fact, similar active species were observed by irradiating KP derivatives; biradicals and carbanion species are formed in the triplet state of KP derivatives [28,29]. The biradicals are resonant structures of the carbanions. Therefore, the proposed photoinitiated polymerization mechanism of the novel thioxanthone derivatives is displayed in Figure 6. The results illustrated that the PBGs could initiate not only the thiol–epoxy polymerization, but also the free radical reactions, such as acrylated polymerization and thiol–ene polymerization [40]. This dual initiation ability makes the PBGs attractive for hybrid polymerization, which usually provides excellent properties for materials.

### 3.5. Dielectric Analysis

**Figure 7:**
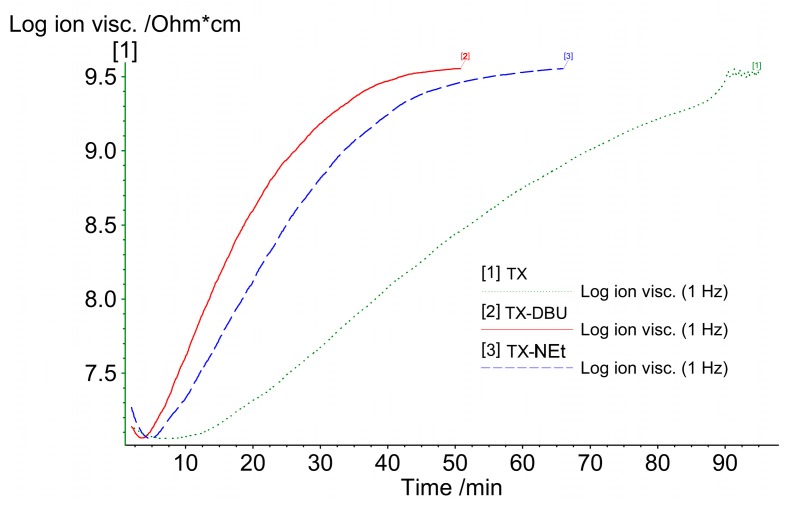
Dielectric analysis curves for photocuring initiated by the PBGs.

The ion viscosity of resin is a useful parameter to trace polymerization processes because of the satisfactory reproducibility of viscosity changes [41]. As a resin mixture cures slowly, a high data sampling rate is necessary. A frequency of 1 Hz was employed for the measurements, and a logarithmic scale was used to ensure that the ion viscosity data are presented clearly (Figure 7). Once upon irradiation, the excited PBGs generated carbanion responsible for the slight decrease in the ion viscosity monitored by the sensor. The linear increase in the log ion viscosity after irradiation resulted from dominant chain growth. The level-off curve indicated the end of the curing process. The TX–catalyzed resin exhibited a slow increase throughout the measurements. The dielectric analysis results revealed that the resin containing TX exhibited the slowest viscosity changes after irradiation due to the relatively inefficient decarboxylation reaction and, therefore, slow chain growth rate. The ion viscosity of the resin catalyzed by TX–DBU increased more rapidly than did that of the resin initiated by TX–NEt. The enhancement should be attributed to the released double active species: carbanion and DBU.

### 3.6. Thermal Properties of Photocured Films

**Figure 8:**
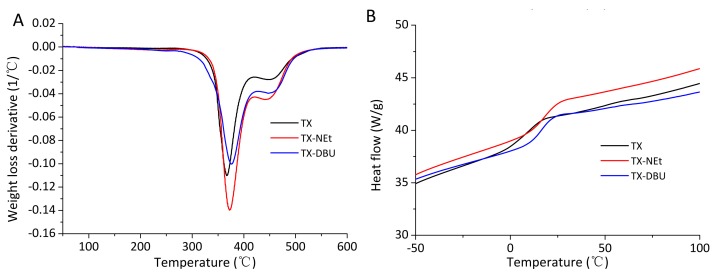
(**A**) DTG curves and (**B**) DSC thermograms of photocured films irradiated by different PBGs.

The photocured films irradiated by the different PBGs were studied by TGA and DSC to study the thermal properties. The thermal stability of the thermosets was determined by TGA under an inert atmosphere. The derivative thermogravimetry (DTG) curves are shown in Figure 8A, and the main parameters are summarized in Table 1. Both of the DTG curves and TG curves (Figure S9) reveal the favorable thermal stability of these films, which were stable up to 298 °C (less than 5% weight loss, *T*_5%_). With a generally two degradation process, all the photocured films gave no significant differences.

Glass transition temperatures (*T*_g_) obtained by DSC are collected in Table 1, and Figure 8B shows the thermograms of the cured films. *T*_g_ obviously increased due to the high activity of novel PBGs resulting from the increase in the conversion of thiol–epoxy monomers. It should be remarked that low *T*_g_ values were observed due to the flexible character of the formed suloether structure [42]. Moreover, the low crosslinking density of the TX containing film exhibited the lowest *T*_g_.

### 3.7. Mechanical Properties of Photocured Films

**Figure 9:**
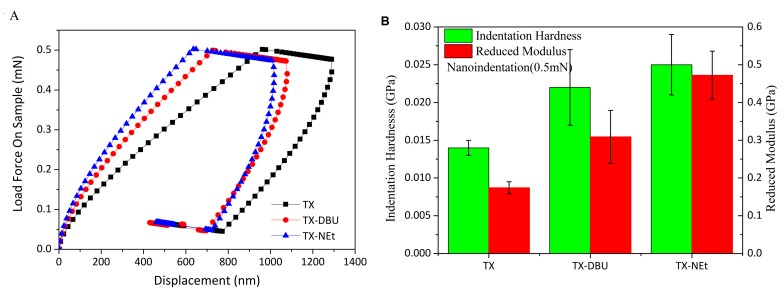
(**A**) Load-displacement curves, and (**B**) reduced modulus and nanoindentation hardness of films subjected to photocuring initiated by the PBGs.

Nanoindentation was used to evaluate the mechanical properties of the films subjected to photocuring initiated by the PBGs. The load–displacement curves for all the investigated films are depicted in Figure 9A. No discontinuities in the curves were observed, indicating that no cracks formed during the nanoindentation tests. The nanoindentation hardness and reduced modulus were determined from the indentation curves and are shown in Figure 9B. The nanoindentation hardness indicates the resistance of the film to surface penetration by an indenter with a force applied to it, and the reduced modulus is related to the Young’s modulus of the test specimen [43]. The photocured films containing TX–NEt and TX exhibited a nanoindentation hardness and reduced modulus similar to those of the films containing TX–DBU, indicating that the efficiency of carbanion-induced photopolymerization is comparable to that of a superbase-catalyzed photopolymerization process. Similar negative stiffness values were observed in the initial period of unloading because the films exhibited identical creep [44]. However, shallower tip penetration for the TX–NEt film was observed. This observation indicated substantial surface hardening caused by the high epoxy conversion of TX–NEt (Figure 9A); this surface hardening was consistent with the efficient carbanion yield and high polymerization rate of the quaternary ammonium salts.

## 4. Conclusions

In summary, we present carbanions as a superbase for catalyzing thiol–epoxy photopolymerization for the first time, and developed novel PBGs to generate carbanions efficiently with the help of quaternary ammonium cations. UV–Vis and photolysis study indicated that the carbanions are produced through a decarboxylation reaction. ^1^H NMR and FT-IR measurement confirmed the carbanion intermediates and its ability of ring-opening polymerization. The results of photopolymerization tests indicated that the generated carbanion exhibited a high polymerization rate and high epoxy conversion compared with traditional superbase DBU. The high initiation ability and straightforward synthesis makes these PBGs promising candidates for commercialization.

## Figures and Tables

**Table 1:** Thermal data of photocured films with different PBGs.

Type of PBGs	*T*_g_ °C	*T*_5%_ °C	*T*_max_ °C
TX	6	328	367
TX–NEt	15	326	372
TX–DBU	18	298	376

## Supplementary Materials

The following are available online at www.mdpi.com/2073-4360/9/9/400/s1, Figure S1: UV-VIS absorbance spectrum of PBGs in acetonitrile solution; Figure S2: UV–Vis spectra changes of TX–NEt solution (10^−4^ M) without (a) and with (b) the addition of phenol red irradiated with an Hg lamp at different light doses; Figure S3: Photos of generated CO_2_ detection: (a) before and (b) for 20 min UV irradiation solution of TX–NEt and TX (5.0 × 10^−3^ M), solutions of Na_2_CO_3_ (2.0 × 10^−4^ M) using an IWATA UV-100 LED irradiating 365 nm light; Figure S4: ^1^H NMR spectrum of TX–NEt in CDCl_3_ with D_2_O under 30 s irradiation; Figure S5: Ring-opening polymerization ^1^H NMR spectra (DMSO-*d*_6_) of BADGE, and mixtures of TX and BADGE irradiated by an LED lamp (A); and the conversion degree of BADGE ring-opening under irradiation (B); Figure S6: Transient optical absorption spectrum following laser excitation (355 nm) of TX–NEt in nitrogen-saturated acetonitrile solution at 25 °C; Figure S7: Transient optical absorption spectrum following laser excitation (355 nm) of TX in nitrogen saturated acetonitrile solution at 25 °C; Figure S8: ESR spectrum of TX–NEt (left) and TX (right); Figure S9: TG cures of photocured films catalyzed by different PBGs.
