# Supplementary material for: Carbanion as a Superbase for Catalyzing Thiol–Epoxy Photopolymerization
Source: Polymers (Basel). 2017 Aug 29;9(9):400. doi: 10.3390/polym9090400 (PMC6418981; doi:10.3390/polym9090400)
Supplement: Supplementary file 1 [file polymers-09-00400-s001.pdf]

Supplementary Materials: Carbanion as a Superbase for Catalyzing Thiol–Epoxy Photopolymerization

Xiaoqing Dong, Peng Hu, Weizhen Shen, Zhiquan Li, Ren Liu, Xiaoya Liu

Experimental Section

Electron Spin Resonance Spin-Trapping

The electron spin resonance (ESR) experiments were carried out using an EMXplus-10/12 X-band spectrometer (Bruker, Karlsruhe, Germany) at 100 kHz magnetic field modulation, which was used to adjust the power intensity to 20 mW. The mixture of PBGs (photobase generators, 1 mol%) and Phenyl-N-tert-butyl-nitrone (PBN, TCI Chemicals, Shanghai, China, 2 mol%) was dissolved in benzene and deoxygenated with nitrogen for 5 min before irradiation. The radicals were generated through photolysis at room temperature (LED source emitting 365 nm light, UV Pro, Shanghai, China, irritation for 30 s).

Laser Flash Photolysis

Laser flash photolysis (LFP) experiments employed the Edinburgh Analytical Instruments F900 (Edinburgh Instruments, Livingston, United Kingdom) which used an Nd/YAG laser (355 nm, 25 mJ per 8 ns pulse) and a multichannel scaler. PBGs samples at concentrations ranging from 0.1 to 0.3 mM were prepared in acetonitrile, which were purged with the appropriate gas (i.e., N_2, Ar_2) for 30 min before, and during, the acquisition of the transient spectrum.

Figures

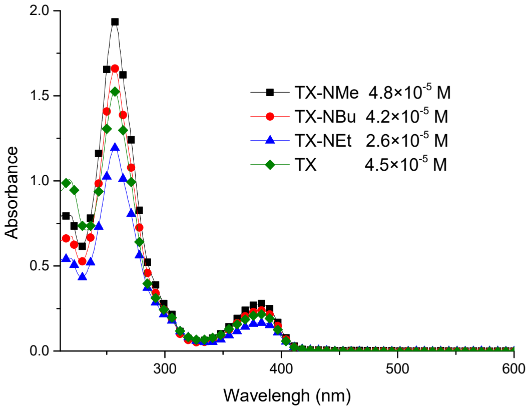

Figure S1. UV-VIS absorbance spectrum of PBGs in acetonitrile solution.

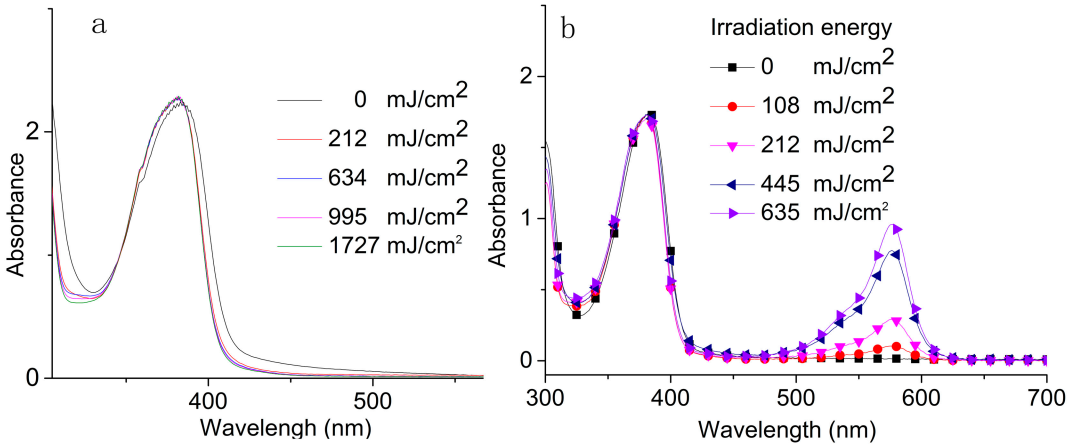

Figure S2. UV-VIS spectra changes of TX-NEt (9-Oxo-9H-thioxanthen-2-yl)-acetatetetraethyl-ammonium) solution (10^{-4} M) without (**a**) and with (**b**) the addition of phenol red irradiated with an Hg lamp at different light doses.

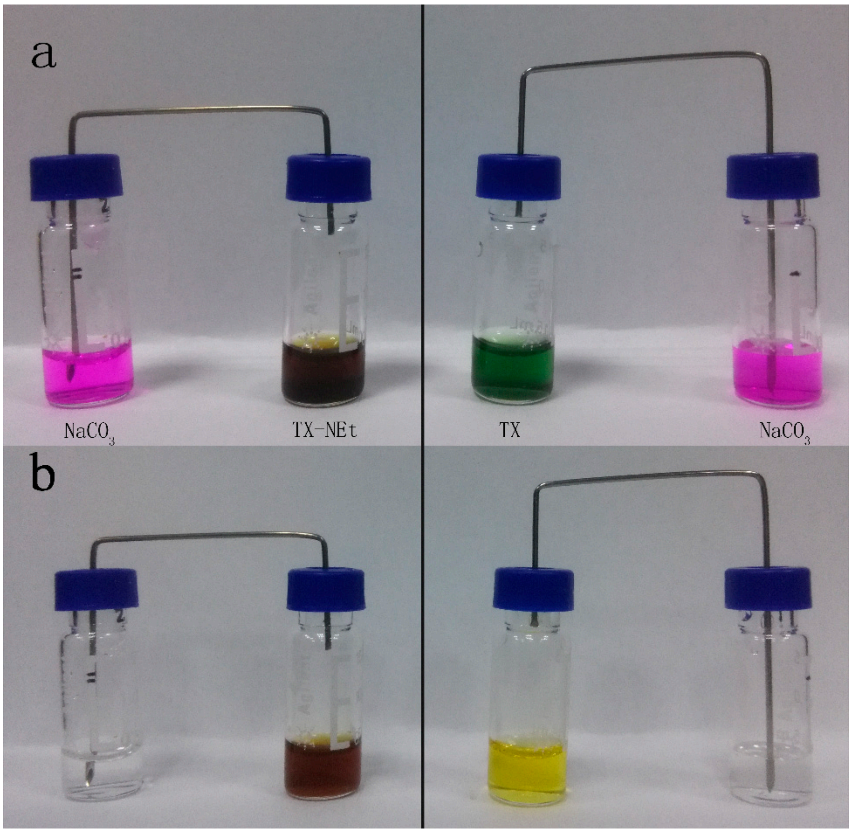

Figure S3. Photos of generated CO_2 detection: (**a**) before and (**b**) for 20 min UV irradiation solution of TX-NEt and TX (thioxanthone acetic acid, 5.0×10^{-3} M), solutions of Na_2CO_3 (2.0×10^{-4} M) using an IWATA UV-100 LED irradiating 365 nm light.

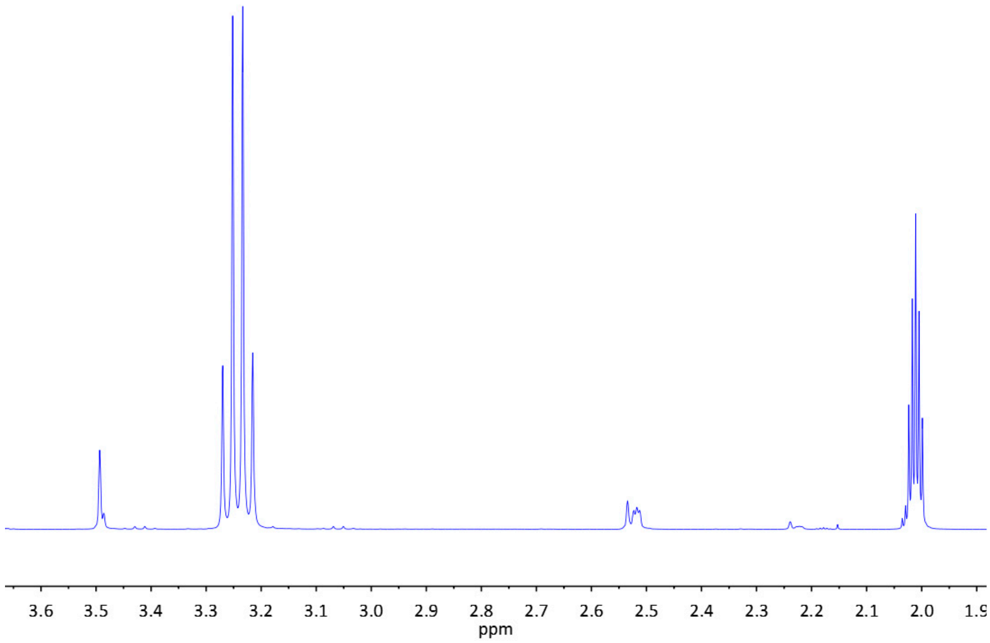

Figure S4. ^{1}H NMR spectrum of TX-NEt in $CDCl_3$ with D_2O under 30 s irradiation.

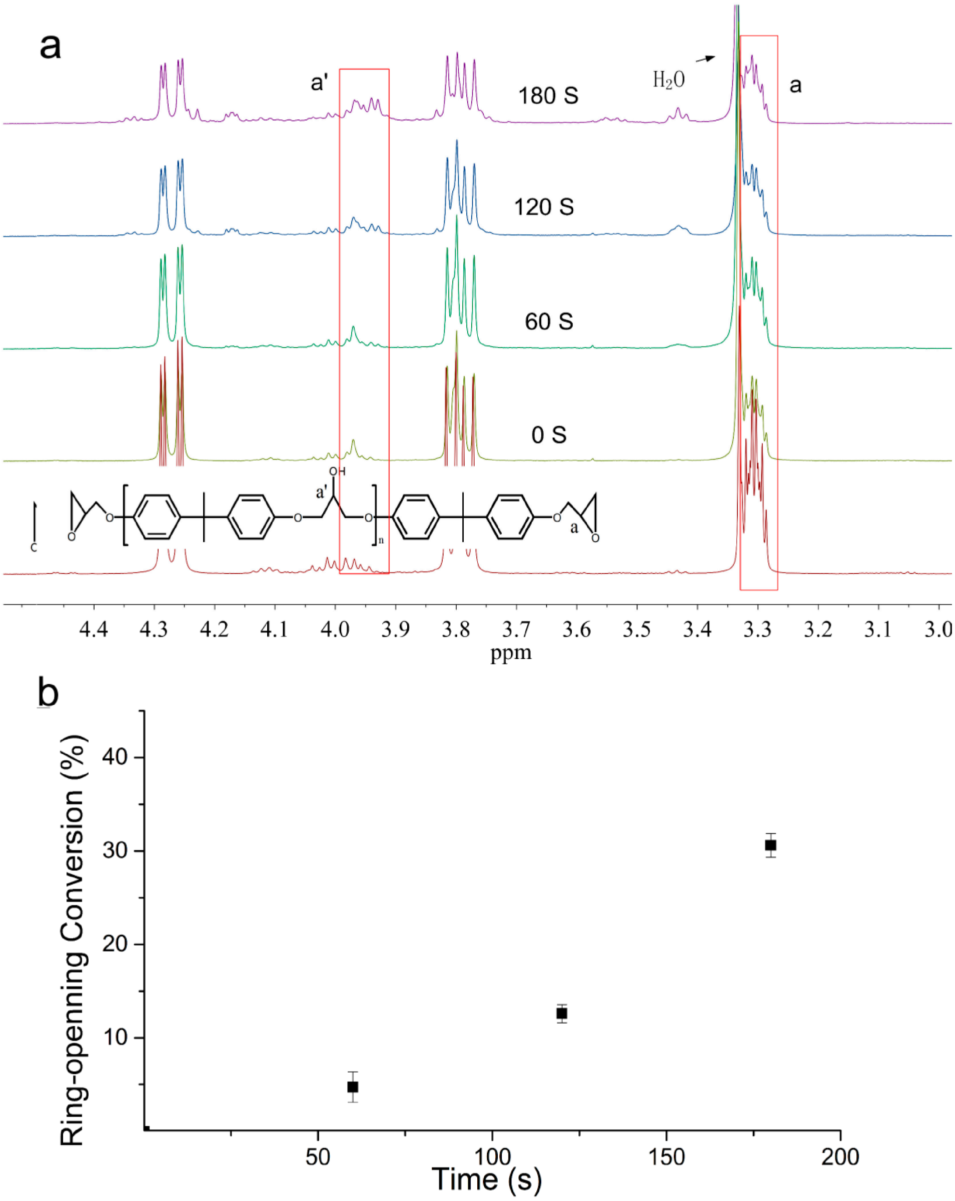

Figure S5. Ring-opening polymerization ^{1}H NMR spectra (DMSO-d6) of BADGE (a diglycidyl ether), and mixtures of TX and BADGE irradiated by an LED lamp (**a**); and the conversion degree of BADGE ring-opening under irradiation (**b**).

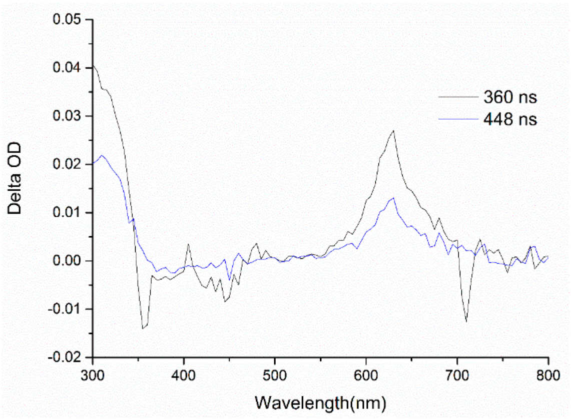

Figure S6. Transient optical absorption spectrum following laser excitation (355 nm) of TX-NEt in nitrogen saturated acetonitrile solution at 25 °C.

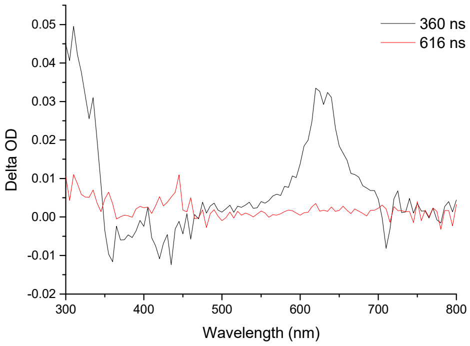

Figure S7. Transient optical absorption spectrum following laser excitation (355 nm) of TX in nitrogen-saturated acetonitrile solution at 25 °C.

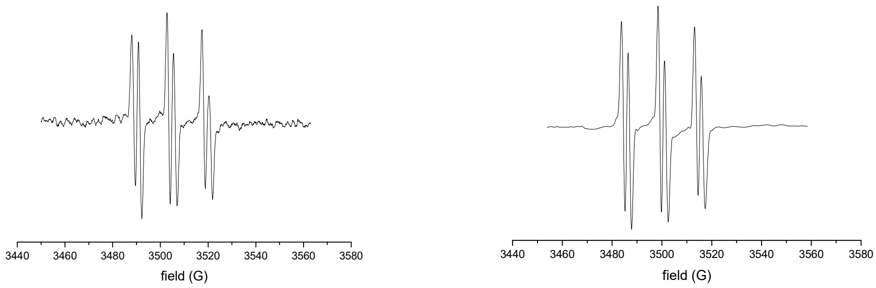

Figure S8. ESR spectrum of TX-NEt (**left**) and TX (**right**).

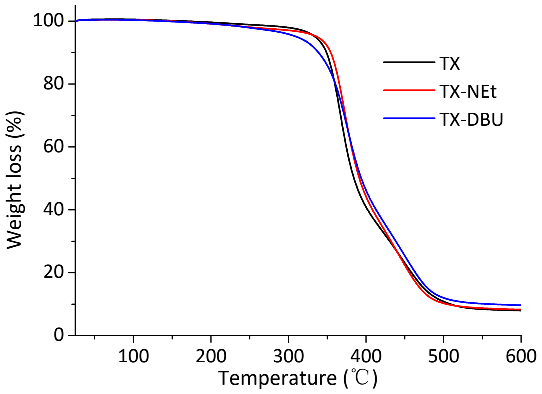

Figure S9. TG cures of photocured films catalyzed by different PBGs.
